# Guidelines for identification and treatment of individuals with attention deficit/hyperactivity disorder and associated fetal alcohol spectrum disorders based upon expert consensus

**DOI:** 10.1186/s12888-016-1027-y

**Published:** 2016-09-22

**Authors:** Susan Young, Michael Absoud, Carolyn Blackburn, Polly Branney, Bill Colley, Emad Farrag, Susan Fleisher, Ges Gregory, Gisli H. Gudjonsson, Keira Kim, Kieran D. O’Malley, Moira Plant, Alina Rodriguez, Susan Ozer, Inyang Takon, Emma Woodhouse, Raja Mukherjee

**Affiliations:** 1 Imperial College London, London, UK; 2 Broadmoor Hospital, West London Mental Health Trust, Crowthorne, Berkshire UK; 3 Children’s Neurosciences, Evelina London Children’s Hospital at Guy’s & St Thomas’ NHS Foundation Trust, Kings Health Partners Academic Health Science Centre, London, UK; 4 Centre for the Study of Practice and Culture in Education, Faculty of Health, Education and Life Sciences, Birmingham City University, Birmingham, UK; 5 Oxford ADHD Centre, Headington, Oxford, UK; 6 CLC Consultancy, Dunkeld, Scotland; 7 Sussex Partnership NHS Foundation Trust, Children & Young People’s Service, Tunbridge Wells, Kent UK; 8 National Organisation for Foetal Alcohol Syndrome-UK (NOFAS-UK), London, UK; 9 Integrated Child Health, Cambridge and Peterborough Foundation Trust, 80 Thorpe Road, Peterborough, PE3 6AP UK; 10 King’s College London, Institute of Psychiatry Psychology and Neuroscience, London, UK; 11 Contracted Medical Writer, San Diego, CA USA; 12 Child and Adolescent Psychiatrist, FASD Specialist, Slievemore Clinic, Dublin, Ireland; 13 President Elect, Intellectual Disability Section Royal Society Medicine, London, UK; 14 University of the West of England, Bristol, UK; 15 National Drug Research Institute Curtin University, Perth, Australia; 16 East and North Hertfordshire NHS Trust, Hatfield, Hertfordshire, UK; 17 East and North Hertfordshire NHS Trust. Centre for Child and Adolescent Mental Health, University College Ibadan, Hatfield, Hertfordshire UK; 18 FASD Specialist Behaviour Clinic, Surrey and Borders Partnership NHS Foundation Trust, Oxted, Surrey UK; 19 Sweden University, Department of Psychology Campus Östersund, Östersund, Sweden

**Keywords:** Attention deficit/hyperactivity disorder (ADHD), Fetal alcohol spectrum disorders (FASD), Co-morbid or associated FASD, Treatment guidelines, Consensus, Interventions

## Abstract

**Background:**

The association of attention deficit/hyperactivity disorder (ADHD) and fetal alcohol spectrum disorders (FASD) results in a complex constellation of symptoms that complicates the successful diagnosis and treatment of the affected individual. Current literature lacks formal guidelines, randomized control trials, and evidence-based treatment plans for individuals with ADHD and associated FASD. Therefore, a meeting of professional experts was organized with the aim of producing a consensus on identification and treatment guidelines that will aid clinicians in caring for this unique patient population.

**Methods:**

Experts from multiple disciplines in the fields of ADHD and FASD convened in London, United Kingdom, for a meeting hosted by the United Kingdom ADHD Partnership (UKAP; www.UKADHD.com) in June 2015. The meeting provided the opportunity to address the complexities of ADHD and FASD from different perspectives and included presentations, discussions, and group work. The attendees worked towards producing a consensus for a unified approach to ADHD and associated FASD.

**Results:**

The authors successfully came to consensus and produced recommended guidelines with specific regards to identification and assessment, interventions and treatments, and multiagency liaisons and care management, highlighting that a lifespan approach to treatment needs to be adopted by all involved. Included in the guidelines are: 1) unique ‘red flags’, which when identified in the ADHD population can lead to an accurate associated FASD diagnosis, 2) a treatment decision tree, and 3) recommendations for multiagency care management.

**Conclusions:**

While clinically useful guidelines were achieved, more research is still needed to contribute to the knowledge base about the diagnosis, treatment, and management of those with ADHD and associated FASD.

## Background

Approximately 5 % of the population world-wide is reported to have a diagnosis of attention deficit/hyperactivity disorder (ADHD) [1], a disorder often associated with co-morbid conditions that can complicate identification and treatment [2]; fetal alcohol spectrum disorders (FASD) are among those conditions that are commonly found to co-exist with ADHD [3]. There is an established knowledge base with regards to identification and treatment guidelines for ADHD, and similarly, yet to a lesser degree, for FASD; there is also an increasing awareness and knowledge base of individuals having both disorders concurrently. There are not, however, formal guidelines for diagnosis, randomized controlled trials of any kind, or evidence-based treatment plans for this unique group of individuals with ADHD and associated FASD [4]. Therefore, this multi-disciplinary meeting was organized to produce identification and treatment guidelines for patients with ADHD and associated FASD according to the clinical expertise and knowledge among the attendees based upon consensus.

In this article we refer to the co-existence of ADHD and FASD as—ADHD and associated FASD—and not FASD with associated or co-morbid ADHD because: 1) most alcohol affected individuals present with and are initially evaluated for attention and executive function deficits [3, 5]; 2) management of ADHD symptoms in the FASD individual is integral to treatment and may minimize the potential impact of the common secondary disabilities associated with FASD [3]; and 3) speculation remains as to the nature of the relationship of the co-existing disorders with each other [3, 6]. The authors recognize that current formal guidelines for the management of ADHD symptoms do not account for the implications of prenatal alcohol exposure in affected individuals, thus the formulation of expanded guidelines for management of ADHD and associated FASD is the focus of this discussion. Firstly, we describe what is known about ADHD and FASD separately and then we describe what is known about the co-existing disorders. Next we discuss how our collaborative efforts led to consensus. Lastly, we present our recommended guidelines for clinicians, with specific regards to: identification and assessment, interventions and treatment, and multiagency liaisons and management. Included are three pertinent case studies that exemplify the need for individualized treatments and services, which commonly extend beyond medical care. A list of abbreviations can be found at the end of the manuscript.

### ADHD

ADHD is a childhood onset, diagnosable neurobiological disorder with genetic and environmental origins [7] characterized by pervasive behavioral symptoms of hyperactivity, inattentiveness, and impulsivity that interfere with functioning and development [8]. In 1968 the American Psychiatric Association officially recognized hyperkinetic impulse disorder, which has since been renamed ADHD in the second edition of the Diagnostic and Statistical Manual of Mental Disorders (DSM-II). Symptoms present relatively early in childhood and persist over one’s lifespan for about half of individuals with childhood ADHD [9, 10]. A systematic review and meta-regression analysis reported an overall worldwide-pooled estimate of 5.29 % [11]. Structural brain MRI studies, comparing individuals with ADHD with controls, report overall reductions in brain volume of those having ADHD and reveal the largest differences in cerebellar regions, the splenium of the corpus callosum, total and right cerebral volume, and right caudate; several frontal regions assessed in two studies also showed large significant differences [12]. In addition to academic and social impairments, children with ADHD are at risk for other psychological disorders and about half have additional behavioral disorders [7, 13]. Meta-analyses of 42 international prison studies, based on data derived from symptom-based clinical instruments, reported 30 % of youth offenders and 26 % of adult offenders have clinically diagnosable ADHD [14]. Furthermore, the adult offenders had higher rates of coexisting psychopathology and greater impairment due to mood, anxiety and personality disorders [15].

Clear diagnostic criteria for ADHD are listed in the Diagnostic and Statistical Manual of Mental Disorders, 5^th^ edition, (DSM-5) [8] and the World Health Organization’s International Statistical Classification of Diseases, 10^th^ edition, (ICD-10) [16] (note that ADHD is the ICD-10 equivalent of hyperkinetic disorder). While the criteria have not changed from DSM-IV [17], DSM- 5 has updated the definition of ADHD to more accurately characterize the experience of affected adults, and examples have been included to illustrate the types of behavior children, older adolescents, and adults with ADHD might exhibit. There is a strong evidence base in the literature regarding guidelines for identification, treatment, and management of individuals with ADHD, and the authors advocate use of the National Institute for Health Care Excellence (NICE) 2008 guidelines [18].

### FASD

FASD is a non-diagnostic descriptive term referring to the full range of diagnosable conditions caused by the deleterious effects of prenatal alcohol exposure (PAE), including neuropsychological, behavioral, and physical abnormalities [19]. The range of clinical phenotypes varies in severity and outcome depending on the level, pattern, and timing of maternal alcohol consumption [19, 20]. Fetal alcohol syndrome (FAS) was first described in 1973 [21]. In 1996 the United States’ Institute of Medicine delineated useful classification terms including FAS, partial fetal alcohol syndrome (pFAS), alcohol related birth defects (ARBD), and alcohol related neuro-developmental disorder (ARND) [22]. It wasn’t until 2000, however, that the term FASD was officially introduced [23].

The implications of heavy PAE are long term and pervasive, affecting the individual, mother, family, and community throughout one’s lifespan [24–26]. FASD are considered a “hidden disability” because most individuals affected by PAE are not identified until adolescence or adulthood, if at all [27]. Although FAS is estimated to occur in 2 to 7 per 1000 live births in young children in the United States, and up to 68 per 1000 in high-risk populations [28, 29], FASD are more prevalent and may occur in as many as 2–5 % of younger school children in the US and some western European countries [30], which means that as many as 6 to 16 million young children in the US may have FASD, based upon the current US population of 320 million [31]. Meta-analyses of children and youth in a child-care system revealed a 6.0 % pooled prevalence of FAS and a 16.9 % pooled prevalence of FASD [32]. A recent study reported a significantly high proportion (86.5 %) of FASD among foster and adopted youth referred to a mental health center, which were previously mis- or undiagnosed [33]. Furthermore, a retrospective assessment of children in the United Kingdom reported prenatal alcohol exposure rates of 34 % among “looked after children” and 75 % among those awaiting adoption [34]. Brain imaging techniques have shown that PAE causes permanent structural alterations to the brain, and reduced overall volume [35, 36]. Studies have demonstrated damage to the corpus callosum, cerebellar vermis, basal ganglia, as well as perislyvian, orbito-frontal, and parietal brain regions of individuals with FASD [35]. Additionally, a review of individuals with FASD reported a pooled prevalence of 90.9 % having abnormal peripheral nervous system function results [37].

There is a high prevalence of co-morbid conditions in individuals with FASD [37]. Long term studies report that adolescents and young adults with FASD have major problems with adaptive behavior, with high rates of disrupted education (61 %), trouble with the law (60 %), confinement (50 %), inappropriate sexual behavior on repeated occasions (49 %), and drug and alcohol related problems (35 %) [26]. Deficits in adaptive functioning can often help clinicians identify individuals with prenatal alcohol exposure [26]. Individuals with FASD present with specific behavioral impairments that make them especially vulnerable to manipulation. They are easily coerced into producing false confessions thereby making them more likely to become involved with the criminal justice system [38]; and one study reported 35 % of individuals with FASD have been in jail or prison at some point [39].

The diagnostic criteria for FAS is well established in the ICD-10 and DSM-5, but the diagnostic criteria for PAE conditions other than FAS are less precise and subject to considerable debate due to lack of available published evidence [40]. Several FASD diagnostic approaches have been designed to promote best practices, but accurate clinical diagnoses remain, for the most part, to be made by expert clinicians who have wider experience with complex neurobehavioral findings. According to the ICD-10 and DSM-5 diagnostic criteria sets, in the absence of confirmed PAE, the signs and symptoms of FAS fall into three categories: 1) a characteristic pattern of facial anomalies (short palpebral fissure, smooth philtrum, thin vermilion border of the upper lip); 2) evidence of growth retardation (pre-and/or postnatal); and 3) evidence of central nervous system (CNS) abnormalities [19–21]. While the presence of facial anomalies enables diagnosis in some children with histories of heavy prenatal alcohol exposure, most PAE affected individuals do not exhibit obvious dysmorphology [41] and/or lack reliable PAE history [42], which greatly hinders identification. Although useful for classification, the IOM’s terms are not listed as diagnosable conditions in the ICD or DSM criteria sets. DSM-5 has proposed a diagnostic criteria set for neurobehavioral disorder associated with prenatal alcohol exposure (ND-PAE), but is not yet intended for clinical use. A recent paper on FASD [43] (which thoroughly elucidates the diagnostic terms and includes the proposed DSM-5 criteria on ND-PAE) and recent book on ARND [44], together explain why a more accessible diagnosis is needed. Until the DSM-5 or ICD-11 have established criteria sets, the authors advocate using the 2005 Canadian Guidelines for the Diagnosis of FASD [20]. These guidelines focus more on the neurobehavioral assessment over growth deficiencies and recommend using a multidisciplinary approach to diagnosis [20].

### ADHD and associated FASD

Individuals with histories of heavy PAE or ADHD are at risk for a wide range of impairments including behavioral and neuropsychological deficits [37, 45, 46]. Children with a diagnosis of ADHD present with impairments similar to those apparent in alcohol-exposed children especially with regards to executive functioning and attention deficits [47]. There are, however, clear distinctions between the two groups; individuals with ADHD with PAE perform worse on conventional tests sensitive to attentional problems and conduct disorder, compared with individuals with FASD [46]. ADHD is the most common psychiatric disorder diagnosed in children with PAE in the United States at a rate of 41 % [48] and in individuals with FASD world-wide at a rate of 48 % [49]. Given the range of a 2–5 % prevalence rate of FASD in the US and some western European countries and the 41–48 % prevalence rate of co-occurring ADHD, we estimate the prevalence of ADHD and associated FASD to be 0.8–2.4 % among children in the US and some western European countries. Although, because many affected individuals are un-diagnosed, we speculate that the two disorders occur together at far higher rates than estimates suggest. Despite the depth of knowledge on the separate disorders and an increasing amount on the concurrence, the exact relationship between the two remains unclear and there is a debate over whether or not the presentation necessarily constitutes separate entities [3, 6, 25]. Furthermore, evidence indicates that ADHD symptoms in individuals identified with FASD may be a specific clinical phenotype [3]. Understanding the relationship between the disorders is complicated by the fact that ADHD is primarily defined descriptively (without a clear etiology) and FASD are primarily defined mechanistically (with a clear etiology). The clinical quality of ADHD in children with FASD often differs from that of children having only ADHD [50]; behavioral studies report exacerbated effects of having both PAE and ADHD compared with alcohol exposure alone [45]. The majority of individuals with PAE lack the physical and facial dysmorphology commonly associated with FAS [41] and many lack reliable histories of PAE [42], which further increases the difficulty in making a clear diagnosis. According to the National Organization of Fetal Alcohol Spectrum (NOFAS), most individuals affected by FASD are often only identified ─ if at all─ subsequent to a referral for learning disabilities or for co-occurring ADHD [27], likely resulting in many mis- or undiagnosed individuals. In the absence of high quality studies examining the relationship between ADHD and FASD and reporting on effective treatments, the authors have produced identification and treatment guidelines for clinicians, based upon expert consensus.

## Methods

Experts from multiple disciplines in the fields of ADHD and FASD convened on the 27^th^ of June 2015 in London, United Kingdom, for a meeting hosted by United Kingdom ADHD Partnership (UKAP; www.UKADHD.com). The meeting included presentations with electronic slides, discussions, and group work; and was recorded and later transcribed. Psychiatrists, neuro-developmental pediatricians, psychologists, education experts, researchers, and others having personal experience with the impact of ADHD and/or FASD, engaged in lively thought provoking discussions throughout the day, with the aim of reaching consensus.

The meeting commenced with five presentations (including case studies) on the following topics, where each was followed by a question and answer session:

- ADHD and FAS: The Clinical Presentation
- Alcohol Exposure and Risk for Development of FAS
- Psycho-pharmacotherapy Interventions for Co-morbid ADHD/FAS
- Education Interventions for Co-morbid ADHD/FAS
- The Criminal Justice Perspective

Following the presentations, all attendees separated into three breakout session groups (smaller subsets of the meeting attendees) to produce a framework of guidelines with specific regards to:

- Identification and Assessment
- Interventions and Treatments
- Multiagency Liaisons and Care Management

The discussions during the breakout sessions were facilitated by group leaders and summarized by note takers. The group leaders then presented their group’s framework of guidelines in a feedback session to all the meeting attendees for another round of discussion and debate. The medical writer consolidated the meeting transcription, electronic slide presentations, and breakout session notes into the manuscript. Lastly, the meeting transcription and manuscript were circulated to all authors for review to ensure agreement and a final consensus for a unified approach to ADHD and associated FASD was reached.

## Results

### Discussion

The authors successfully produced identification and treatment guidelines for children and adults with ADHD and associated FASD, to aid clinicians in caring for this unique patient population, and the results follow below. Due to the nature of the co-existing disorders, it was recognized that services beyond medical care are required for the vast majority of patients.

## Identification and assessment guidelines

When considering the possibility of ADHD co-existing with FASD it is essential to investigate, with hopes of obtaining, an accurate and detailed PAE history. It is also important to routinely screen for FASD when ADHD is identified or for ADHD when FASD is identified; the DSM-5 and ICD-10 criteria sets are to be utilized for diagnosing ADHD and FAS, and we advocate the use of the 2005 Canadian guidelines for diagnosing all other FASD until the DSM-5 and ICD-11 criteria sets are further established. It is important to recognize the unique characteristics or ‘red flags’ that may be present in the individual with ADHD, which may assist in the identification of co-existing FASD. Prevalence rates of ADHD are known to be high in certain high-risk populations such as prisoners, ‘looked after’ children, adoptees, and individuals born prematurely. Efforts should be made among medical, social, educational, and criminal justice services to increase awareness of the need for appropriate referrals for individuals in these high-risk groups. They should be referred to suitable local pathways for either ADHD or FASD, based upon their primary presenting condition. It is recognized that individuals with subtle presentation may fail to meet the threshold for ADHD and are likely to be missed with this approach; ideally, the development of locally based complex neuro-developmental services is recommended. Neuro-developmental services could help identify and further assess affected individuals, which could potentially minimize the secondary health and social disabilities associated with ADHD and FASD.

## The clinician should carefully identify and assess

### PAE history

Maternal alcohol use during pregnancy should be included in a standard interview for those being assessed for ADHD. The World Health Organization’s Alcohol Use Disorders Identification Test offers a simple method of screening for excessive drinking and assists in making a brief assessment [51]. It is recognized that clinicians, patients and their families may be uncomfortable with this line of questioning; therefore it is recommended that enquiries concerning possible PAE be incorporated in the context of obtaining a detailed family history, in order to ‘normalize’ the questioning for both the clinician and the individual under assessment. Additionally, it is recognized that mothers may find difficulty in admitting retrospectively that their child suffered PAE, possibly as a result of perceived stigma. It is also important to ascertain the accurate amount of alcohol consumed during pregnancy—depending on the size and alcohol content of the drink, two drinks per day could equate to two or more units. Obtaining an accurate alcohol use history will likely depend upon the interviewer’s skill and training. As a guide, the clinician should investigate typical pre-pregnancy lifestyle factors including general drinking behaviors, use of prescription medication, and other drugs. As part of this investigation the clinician may need to access historical records from social workers, midwives, and/or obstetricians. The lack of honest and accurate estimates of alcohol consumption combined with the oft-times lack of availability of the mother [52] is problematic and can result in large groups of potentially high-risk individuals remaining unidentified. Best practice therefore suggests that countries adopt standardized investigative procedures to identify and track these potentially at risk individuals and their families. Due to the sensitive nature of obtaining an accurate history of drinking during pregnancy, an empathic, non-judgmental approach to questioning is essential.

### ADHD or FASD history and additional screenings

If the child or adult has been previously diagnosed with ADHD then the clinician should routinely screen for FASD and likewise FASD for ADHD. A diagnosis of FAS should be made according to DSM-5 and ICD-10 criteria sets and all other FASD should be assessed using the 2005 Canadian guidelines. A diagnosis of ADHD should be made according to DSM-5 and ICD-10 criteria sets, utilizing standard screening and diagnostic tools: Conners’ Rating Scales; Swanson, Nolan, and Pelham-IV (SNAP-IV); and ADHD Rating Scale-IV. It is recommended that the affected individual be referred, when appropriate, to neuro-developmental services for additional assessments in adaptive behavioral functioning, executive function, and communication abilities.

### Unique characteristics of ADHD with associated FASD

We recognize a number of unique characteristics or ‘red flags’, which when present in individuals with ADHD, may assist in the identification of associated FASD. These ‘red flags’ can be considered as potential risk factors for having FASD. Clinicians are to be alerted if any of the following unique characteristics are identified in patients with ADHD:

- Possible or confirmed history of maternal alcohol consumption during pregnancy,
- Predominant presentation with inattentive subtype of ADHD, in addition to some impulsive behaviors,
- Failure to respond to, or increased behavioral disturbance when prescribed methylphenidate,
- Poor psychostimulant response in children with IQ under 50,
- Physical indicators of FAS or pFAS, including delayed growth and/or specific facial features (e.g., smooth philtrum, thin upper lip, and small eye openings), and/or a
- Lack of response to typical behavioral interventions.

## Intervention and treatment guidelines

**Fig. 1:**
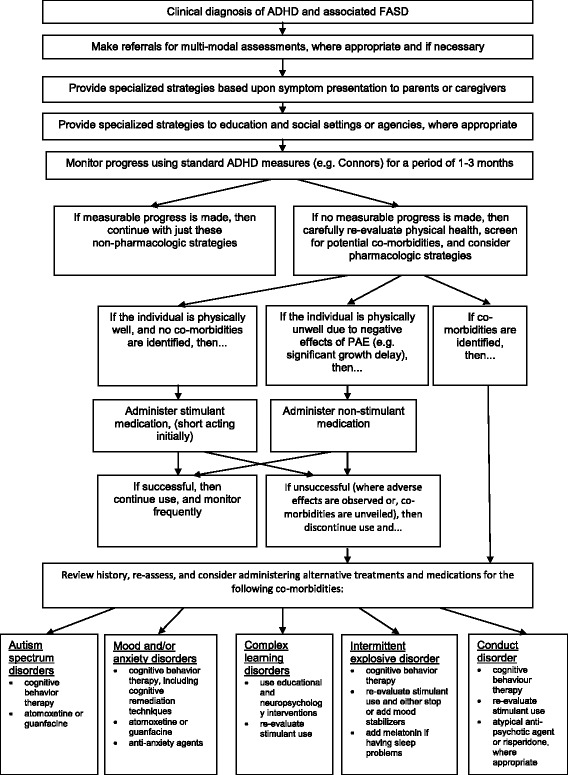
Treatment Decision Tree

Treatments for ADHD and associated FASD require an individualized approach, as do all neuro-psychiatric disorders. Clinicians should refer for multi-modal assessments (often involving parents, teachers, occupational therapists, and speech and language therapists), when necessary, to assist them in treatment plan development. The Centers for Disease Control offers guidelines to service providers in making referral decisions specific to individuals with FAS [41], but can be a good resource for referral considerations for individuals with ADHD with associated FASD. When developing the treatment plan the primary rearing environment should be recognized and evaluated because it has different modulating effects on affected individuals. The following co-occurring problems must be considered as contributors to the clinical presentation; early onset post-traumatic stress disorder due to direct or indirect exposure to violence including specific alcohol related violence, and/or reactive or disorganized attachment disorders due to problems with adoptions or multiple care givers (as in sharing parenting arrangements between foster and birth families). Furthermore, because a combination of non-pharmacological and pharmacological treatments are most often required when treating ADHD, it is recommended that this also be the case for individuals with associated FASD—noting that pharmacological treatments should ideally be administered secondary to non-pharmacological (psychological and behavioral) therapies. While treatments differ between ADHD with and without associated FASD, the 2008 NICE treatment guidelines for ADHD are mostly applicable to this group and should be used as a basis on which to build the treatment plan and the medication options available for ADHD are to be considered in the context of PAE. If other co-morbidites are unveiled throughout the assessment process, then it will be necessary to modify the treatment plan. NICE guidelines allow for treatment with medication after 5 years of age following psychological and behavioral therapies and state that medication can be considered as the first option for individuals with a severe presentation of ADHD. Individuals with ADHD and associated FASD (where hyperactivity, rather than a sensory seeking need, is a predominant feature) will likely be categorized as having a severe presentation of ADHD and therefore may be treated with medication as a first option upon diagnosis. Because ADHD and associated FASD affects individuals at a very young age with symptoms persisting over their lifespan, it is crucial that they are treated, monitored, and supported throughout their entire life. Additionally, it is important to maintain a non-judgmental attitude towards affected individuals and their families while developing and delivering the suitable treatment plan. We developed a treatment decision tree (Fig. 1) unique to children, adolescents, and adults with ADHD and associated FASD that outlines and supplements our guidelines.

## The clinician should administer the following treatments

### Non-pharmacological treatments for the family

Family support is usually necessary, and oftentimes special support may be required for the birth mother, because she may need therapy to cope with possible feelings of guilt and shame relating to alcohol use during pregnancy. Educating and encouraging the birth mother may result in reduced or no alcohol use in subsequent pregnancies. It is also important to recognize the particular needs of adoptive parents and additionally to recognize the ongoing impact of the child’s condition on the family because they may need support groups or respite services.

### Non-pharmacological treatments for the child

Treatment should be delivered primarily via the parents and/or care givers by educating them in healthcare and child training strategies, and techniques about the best ways to care for and help their child. It is important for affected children, and especially for those with additional co-existing learning difficulties, to be fully assessed. It may be necessary to develop a formal individualized educational plan to access additional services to address the need for educational supports.

### Non-pharmacological treatments for the adolescent and adult

Treatment can be delivered directly to the individual. With regards to treatment decisions, however, it must be first established whether or not the individual has the capacity to make such decisions and whether or not it is appropriate to have the family involved as part of a shared plan or agreement. Safeguarding issues need to be taken into account when dealing with children, adolescents, and vulnerable adults. These individuals are capable of attending and benefiting from group treatment/therapy sessions. Symptoms and their associated impairments can become more marked in older children as they progress into adulthood, thus key topics that should be included in therapy, group or otherwise, are: risk taking behaviors, inappropriate sexual behaviors, problem solving skills, social skills, and sleep hygiene techniques. It is of high importance to note that risk taking behaviors and problem-solving deficits in combination with impulsivity may increase the risk of suicide.

### Pharmacological treatments when psychological and behavioral interventions are inadequate

It is important to carefully monitor progress and measure the effects of non-pharmacological treatments through goal-based outcomes. If the co-existing disorders are still causing significant impairment, after a short period of one to three months, then a progression to the use of medication should be made. It is recognized that family involvement with regular and frequent follow-up visits are essential to successful pharmacological treatment. If a child resides within a chaotic family it will be especially necessary to assess whether a responsible adult is correctly administering the medication.

### Pharmacological treatments following baseline physiological tests

The need to physiologically assess the individual and run baseline tests is emphasized as highly important for this group. The NICE treatment guidelines for ADHD recommend the monitoring of blood pressure, pulse, height, weight, and the investigation of cardiovascular history with an ECG prior to medication use. The following additional baseline tests should ideally be ordered, to investigate the possible damaging effects of PAE: full blood count (FBC), thyroid function test (TFT), liver function test (LFT), blood sugar, folate, gamma-glutamyltransferase (GGT; a test specific to alcohol, especially important for teenagers), blood pressure, and renal function test (RFT); these baseline tests should be repeated if indicated clinically. Additionally, care should be taken to obtain a full clinical seizure history, if indicated clinically.

### Pharmacological treatments to improve attention and reduce hyperactivity and impulsivity

**Fig. 2:**
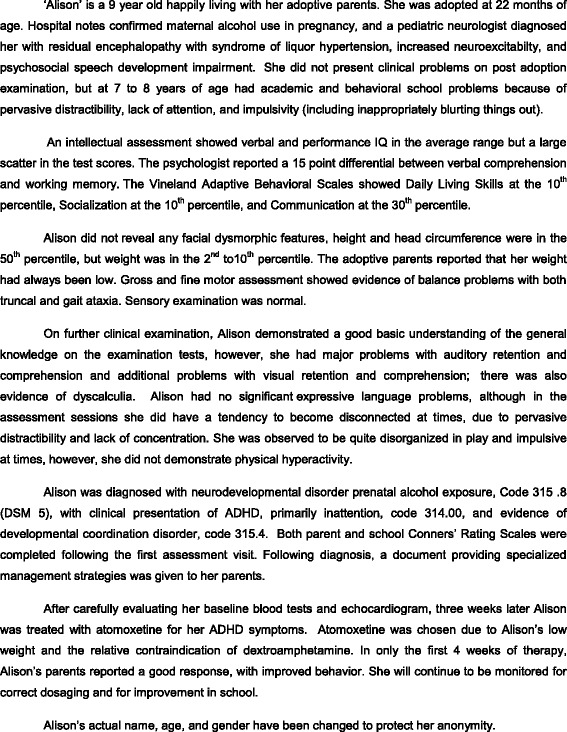
Case Study 1—Pharmacological Treatment

Currently developed medication can treat the symptoms of ADHD, but cannot cure the disorder. Medication can improve attention and reduce hyperactivity and impulsivity, thereby allowing the individual to effectively manage their difficulties, access and engage with suitable therapies, and build upon their skills. Management of the core symptoms may improve learning, minimize their potential impact, and ameliorate the common secondary disabilities associated with FASD. Case study 1 (see Fig. 2) reveals the complex nature of the assessment process and demonstrates how a carefully managed treatment plan can have a positive impact on affected individuals.

### Pharmacological treatments with careful regards to dosage and side effects

The side effects of medication use, among individuals with ADHD and associated FASD, may be similar when compared with individuals with ADHD, but more marked due to the abnormal physiological aspects of the disorder. Some stimulant medications can lead to more pronounced anxiety, appetite, and sleep problems; they may also affect growth hormones and may increase the prevalence of tics. The response to medication can be idiosyncratic and unpredictable because some medications may cause a regression of behavior. In particular, high doses of atomoxetine (ATX) may cause liver damage, therefore low doses are recommended initially. Before increasing a medication dosage it is recommended that the non-pharmacological strategies are revisited and additional assessments are made to account for any under or misdiagnosed co-existing conditions. The individual should be re-evaluated and monitored frequently throughout the intervention process.

### Pharmacological treatments with careful regards to the risks of developing an addictive disorder

Short-acting stimulants are typically trialed first in patients with ADHD to examine tolerance before progressing to long-acting stimulants. This treatment can be problematic, however, in adolescents with ADHD and associated FASD because short-acting stimulants may potentially contribute to the development of addictive disorders during the teenage years. Therefore, long-acting/biphasic stimulants or atomoxetine (which do not give an immediate ‘buzz’) are recommended because they are less likely to contribute to the development of an addictive disorder.

## Multi-agency liasons and care management

The effects of ADHD and associated FASD are lifelong and pervasive, affecting the individual, mother, family, and community. Therefore, it is extremely important that interventions are comprehensive, continue throughout the lifespan, and extend beyond medical care to include when necessary: social, educational, mental health, employment, and criminal justice services. These interventions are likely to be productive when service agencies collaborate with each other. The coordinated efforts of service professionals should support not only affected individuals, but their families as well. In addition to multi-agency collaboration, it is important that each agency offers distinct services designed to enhance quality of life and long term outcomes. While the non-pharmacological treatments previously mentioned cover some of these aspects, there are many additional services that may need to be accessed and managed at different phases of life.

## The clinician should make referrals for the following services

### Social services for the family

If there is suspected or confirmed PAE and/or subsequent failure to engage with professionals, social service referrals for the mother and developing child may need to start prior to birth. Because affected children often display challenging behaviors, social service professionals can offer parents, caregivers, and siblings, training and/or support for managing those behaviors, thereby bolstering family resilience. Family resilience is also likely to be enhanced with the provision of respite services and group support. Social service professionals should routinely review the needs of the family, including possible financial needs, in order to provide timely and comprehensive services, which is especially important during key transition times.

### Social services for the young child

Young children would benefit from preschool placements, where staff have an understanding of FASD and ADHD, and can offer an appropriate environment to meet the developmental needs of the child; and may be referred for specialized stimulating play therapy and/or other preschool services. It may be required that pediatric medical, mental health, social care, and educational professionals coordinate their services (with parental input) to ensure coherent and consistent support. It is important to recognize the potential need for intervention by local authorities or the need for a referral to an adoption specialist. Given the high incidence of placement breakdown, and the subsequent impact of repeated failure on the child and care system, commissioning officers should carefully evaluate the relative merits of foster and adoption placements against more specialized and perhaps resilient services. When children with ADHD and associated FASD are separated from their birth mothers and moved through the care system, they are often inaccurately identified as having insecure or disorganized attachment disorders, instead of being accurately identified as having developmental, emotional, and behavioral difficulties attributed to PAE. In addition, safeguarding issues should be assessed and, in cases of abuse and exploitation, child protection services should be informed and accessed.

### Social services for the adolescent

Social services may need to be accessed because puberty is a particularly vulnerable phase of development. Many young people affected by PAE may crave friendship and a sense of belonging, but often lack the skills required to achieve these. Supported social groups may therefore assist in establishing meaningful relationships with peers within the local community, which is thought to be a protective factor in averting deterioration in mental health, and enhance quality of life. It is important to recognize breakdowns and/or transitions in living accommodations, education placement and treatment plans for these young adults. It is also important to recognize the increased risk of drug and alcohol abuse or misuse during adolescence. The clinician may have a key role regarding safeguarding issues by screening for abuse and exploitation, and accessing child protection services if necessary.

### Educational services for the preschool child

Additional support for the child may be necessary starting in preschool. It is important to recognize the need for child friendly, age-appropriate tasks and activities. It is also important that young children receive broad assessments of needs across multiple measures, and particularly in language comprehension. The pre-school and young primary school child, and family, may benefit from home-visits from educational services to help parents to understand the developmental benefits of learning through play, methods for encouraging and enhancing verbal and non-verbal communication, and in managing challenging behaviors.

### Educational services for the primary school child

It is important to obtain a comprehensive assessment of needs and functional analysis of behavior, and to consider the possible impact of deprivation and limitations unique to these children. It may be necessary to make a referral for the development of an individualized education plan with track-able goals (key learning and developmental targets attainable within a specified time-frame) to document progress. Education planning should include input from students themselves, parents and/or caregivers, and teaching professionals. The assessments and individualized education plan are helpful in determining the student’s best learning environment (e.g., inclusive mainstreaming or special education) and may be required to access education supports and appropriate placement. It is essential for all involved to recognize that these students often benefit most from a strengths-based approach and from differentiated learning. Teachers and educational psychologists will most likely require training to develop relevant assessments and educational strategies specific to this population. Staff awareness, understanding, and competence are key factors in a student’s progress.

**Fig. 3:**
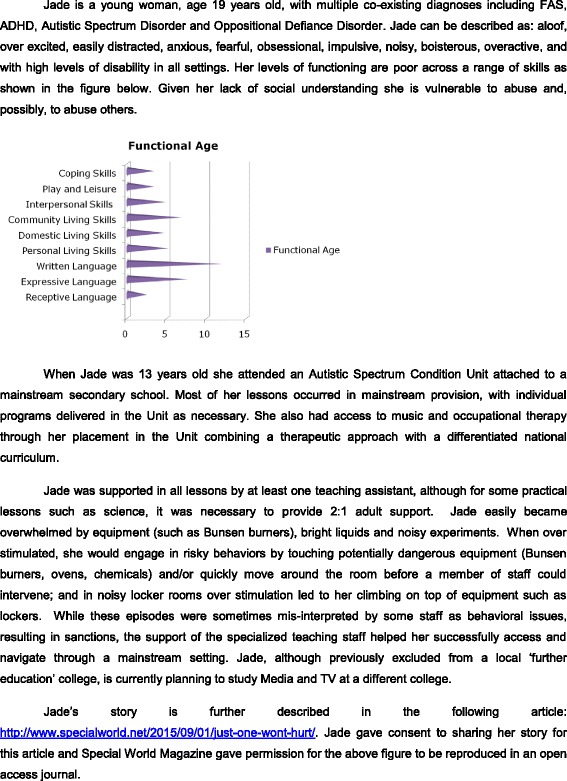
Case Study 2—Secondary Education Provision

Teaching professionals need to consider not only the curricular and staffing requirements of affected students, but also their sensory and social needs. Teachers and support staff need to identify the student’s possible barriers to effective engagement with peers (e.g., social communication and an understanding of ‘friendship’), and provide supportive environments in which social skills may be developed or enhanced. Individuals having difficulty with arousal and mood self-regulation may find mainstream settings too overwhelming and may benefit from a more supported special education setting. Nevertheless, even severely affected individuals can be successfully mainstreamed with support (see case study 2; Fig. 3). Challenging behaviors and/or a disengagement from learning often arise when students are not placed in the appropriate setting. Teachers and support staff should assess challenging behavior from a functional, rather than judgmental, perspective and be sensitive to the potential impact that their own responses may have on a student when difficulties arise. Punitive measures are less likely to be effective than strategies based on positive behavior management.

### Educational services for the adolescent and adult

Educational supports are likely needed when there are breakdowns and/or transitions in placement in secondary school or higher education and when the individual shows behavioral signs of being disengaged from schooling. Because school is a moderating influence, the premature cessation of formal learning is likely to elevate the risk of offending behavior, which can result in a decline in long term mental health. Furthermore, family resilience is also negatively impacted by the school exclusion or placement breakdown. Educational planners must therefore be responsive to the dynamic nature of student’s individual needs and make appropriate provisions.

### Employment services

Affected teenagers and adults will likely require help in finding and maintaining employment, but with proper support can engage in meaningful employment. Employment services should be aimed at enhancing skill development, thereby increasing job prospects and sustainable engagement in work. Post-school transition plans should consider the educational and developmental level (rather than chronological age) of the young person. Affected individuals may require staged transitions into employment via work experience, supported with mentors or job coaches, and college link courses in order to fulfill their vocational potential. The identification of specific barriers (e.g., in establishing effective work-place relationships) may be more beneficial than the provision of generic support.

### Criminal justice services

The clinician may have a role in helping affected children or adults by recommending an appropriate representative to interface with the criminal justice system and/or by referring for the development of an identification card indicating the presence of a disability that the affected individual can present to criminal justice service personnel if necessary. According to the National Organization on Fetal Alcohol Syndrome, “The criminal justice system can help FASD-affected individuals by: educating judges, lawyers, and parole officers about the characteristics and behaviors of individuals with FASD, establishing a screening and referral process for those with ADHD and FASD who enter the juvenile justice or adult criminal justice system, establishing/utilizing alternative sentencing programs for affected individuals who have committed non-violent offenses, offering referral information for the children of incarcerated women who may have been prenatally exposed to alcohol.” [27]. These affected individuals should be diverted, whenever appropriate, into interventional services with the goal of support and skill development to improve functioning in society.

**Fig. 4:**
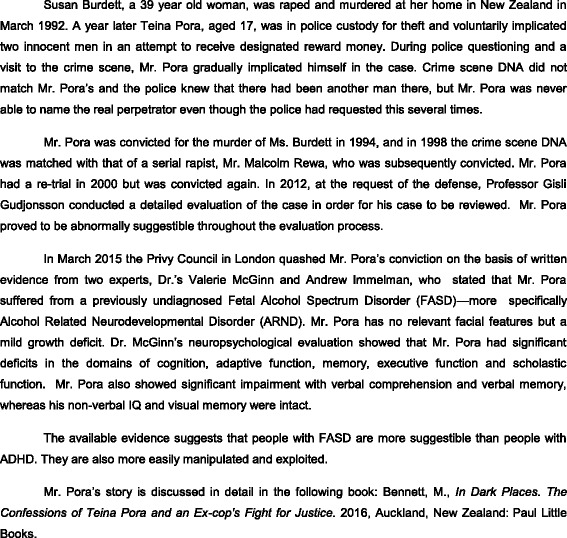
Case Study 3—Criminal Justice System

Case study 3 (see Fig. 4) illustrates how a failure to recognize and diagnose FASD could result in a miscarriage of justice. People with FASD are likely to require the services of a ‘registered intermediary’ in court to facilitate effective communication, and in some cases also during police questioning. As a minimum, like young persons with ADHD, they should have an ‘appropriate adult’ present during police interviews in order to ensure fairness and justice [53].

## Consensus and Conclusions

Although more research is needed to identify, treat, and manage affected individuals, the authors successfully came to a consensus for a more unified approach to ADHD and associated FASD. The authors agreed that there is a need to increase the number and training of clinicians and to expand services for this unique population. The authors also agreed upon the need to develop complex neuro-developmental services that could help identify and further assess affected individuals, and intervention services that could help divert affected individuals away from the criminal justice system, when appropriate. It is likely that governmental support will be necessary in the development of these services.

The most significant outcome of the collaborative efforts amongst the multi-disciplinary professionals was the production of clinically meaningful guidelines, based upon consensus. Included in the guidelines are unique ‘red flags’, which when identified in the ADHD population can lead to an accurate associated FASD diagnosis, a treatment decision tree, and recommendations for multi-agency care management. Given the lifelong, pervasive nature of the associated disorders, the authors’ emphasized that a lifespan approach to treatment needs to be adopted by all involved. The implementation of these guidelines has the potential of making a significant, positive impact on affected individuals.

## Abbreviations

ADHD: Attention deficit hyperactivity disorder
ARBD: Alcohol related birth defects
ARND: Alcohol related neuro-developmental disorder
CNS: Central nervous system
DSM: Diagnostic and statistical manual of mental disorders
FAS: Fetal alcohol syndrome
FASD: Fetal alcohol spectrum disorders
ICD: International classification of diseases
IOM: United States’ institute of medicine
ND-PAE: Neuro-developmental disorder associated with prenatal alcohol exposure
NICE: United Kingdom’s national institute for health care excellence
NOFAS: United States’ national organization of fetal alcohol syndrome
PAE: Prenatal alcohol exposure
pFAS: Partial fetal alcohol syndrome
